# Expression of CD38 on Macrophages Predicts Improved Prognosis in Hepatocellular Carcinoma

**DOI:** 10.3389/fimmu.2019.02093

**Published:** 2019-09-04

**Authors:** Jian Hang Lam, Harry Ho Man Ng, Chun Jye Lim, Xin Ni Sim, Fabio Malavasi, Huihua Li, Josh Jie Hua Loh, Khin Sabai, Joo-Kyung Kim, Clara Chong Hui Ong, Tracy Loh, Wei Qiang Leow, Su Pin Choo, Han Chong Toh, Ser Yee Lee, Chung Yip Chan, Valerie Chew, Tong Seng Lim, Joe Yeong, Tony Kiat Hon Lim

**Affiliations:** ^1^Division of Pathology, Singapore General Hospital, Singapore, Singapore; ^2^A. Menarini Biomarkers Singapore Pte Ltd., Singapore, Singapore; ^3^Duke-NUS Medical School, Singapore, Singapore; ^4^Translational Immunology Institute (TII), SingHealth DukeNUS Academic Medical Centre, Singapore, Singapore; ^5^Temasek Polytechnic, Singapore, Singapore; ^6^Department of Medical Science, University of Turin and Fondazione Ricerca Molinette, Turin, Italy; ^7^Division of Medicine, Singapore General Hospital, Singapore, Singapore; ^8^Centre for Quantitative Medicine, Duke-NUS Medical School, Singapore, Singapore; ^9^Division of Medical Oncology, National Cancer Centre Singapore, Singapore, Singapore; ^10^Department of Hepatopancreatobiliary and Transplant Surgery, Singapore General Hospital, Singapore, Singapore; ^11^Institute of Molecular Cell Biology, Agency of Science, Technology and Research (A^*^STAR), Singapore, Singapore

**Keywords:** macrophage, CD38, marker, prognosis, cancer, hepatocellular carcinoma

## Abstract

**Background:** CD38 is involved in the adenosine pathway, which represents one of the immunosuppressive mechanisms in cancer. CD38 is broadly expressed across immune cell subsets, including human macrophages differentiated *in vitro* from monocytes, but expression by tissue-resident macrophages remains to be demonstrated.

**Methods:** Tissue samples were obtained from 66 patients with hepatocellular carcinoma (HCC) from Singapore and analyzed using immunohistochemistry. Tumor-infiltrating leukocytes (TILs) were further examined using DEPArray™, and the phenotype of freshly isolated TILs was determined using flow cytometry.

**Results:** CD38 was frequently co-expressed with the macrophage-specific marker CD68. CD38^+^CD68^+^ macrophage density was associated with improved prognosis after surgery, while total CD68^+^ macrophage density was associated with poor prognosis. DEPArray™ analysis revealed the presence of large (>10 μm), irregularly shaped CD45^+^CD14^+^ cells that resembled macrophages, with concurrent CD38^+^ expression. Flow cytometry also revealed that majority of CD14^+^HLA-DR^+^ cells expressed CD38.

**Conclusion:** CD38 expression was clearly demonstrated on human macrophages in an *in vivo* setting. The positive association identified between CD38^+^ macrophage density and prognosis may have implications for routine diagnostic work.

## Introduction

Human hepatocellular carcinoma (HCC) is the most common primary malignancy of the liver (1, 2), and represents a severe, worldwide threat to human health and quality of life. Patient survival after surgery remains relatively low, with 5-year survival rates after resection for early-stage disease ranging between 17 and 53%, and recurrence rates being as high as 70% (2–4). Therefore, it is important to identify biomarkers that reliably distinguish patients at high risk of recurrence. HCC progression is known to be driven by chronic inflammation, which may arise from viral infection, hemochromatosis and alcoholic or non-alcoholic steatosis (5). The innate immune system also appears to serve a key role in the progression of HCC. For example, tumor-associated macrophages (TAMs) are widely considered to be correlated with poor prognosis in patients with HCC (6–9).

CD38 is a multifunctional, membrane-associated ectozyme belonging to the ribosyl cyclase family, and is widely expressed across the immune system. This molecule was initially discovered on the surfaces of thymocytes and T cells. Subsequently, it was found to be expressed by other types of immune cell and certain non-lymphoid tissues, including brain, eye, gut and prostate tissues (10). CD38 cleaves NAD^+^ and NADP^+^ to generate the Ca^2+^-mobilizing compounds adenosine diphosphate ribose (ADPR), cyclic ADPR (cADPR), and nicotinic acid adenine dinucleotide phosphate (NAADP) (10). It also functions as a receptor, mediating the transduction of signals associated with activation and proliferation. These dual enzymatic and receptorial functions mean that CD38 is involved in a diverse range of cellular activities. In the context of the immune system, CD38 is known to induce pro-inflammatory and regulatory cytokine production in monocytes and dendritic cells, and to induce proliferation and inhibit apoptosis in lymphocytes (11). Downregulation of CD38 alters the migration of neutrophils and monocytes (12), and impairs the innate immune response against *Listeria monocytogenes* infection (13). CD38 is also known to be present in monocytes, where it acts as a co-accessory molecule for MHC Class II-induced T lymphocyte activation by superantigen (14).

In addition, CD38 is involved in the adenosinergic pathway via its NADase function. This pathway is of significant interest to the field of cancer immunotherapy, as it represents a major alternative immunosuppressive mechanism to the PD-1/PD-L1 pathway. Here, the CD38-CD203a (also known as ENPP1 or PC1)-CD73 salvage pathway generates adenosine through the degradation of pyridine metabolites, such as NAD^+^. Specifically, CD38 hydrolyses NAD^+^ to ADPR, and CD203a further degrades ADPR to adenosine monophosphate (AMP). Following this, CD73 dephosphorylates AMP to adenosine (15–17). Tumor proliferation, survival, adhesion and migration may be regulated through the adenosine pathway. For example, in immune cells, adenosine molecules hamper vital effector cell functions and may be involved in mediating T cell exhaustion (18). A recent study using a murine lung cancer model reported that CD38 expression on cancer cells was upregulated in response to PD-1/PD-L1 blockade, resulting in the inhibition of CD8^+^ T-cell function via adenosine receptor signaling (19).

Our group recently established the relevance of CD38 to HCC by identifying a correlation between CD38^+^ tumor-infiltrating leukocyte (TIL) density and improved patient survival (20). We studied the expression of CD38 on lymphocytes, Natural Killer (NK) cells, NKT cells and monocytes, but not on macrophages. Indeed, CD38 expression has previously been reported on all major leukocyte populations, including B cells, T cells, NK cells, monocytes and dendritic cells (10). However, with respect to macrophages, data concerning CD38 expression is limited. Upregulation of CD38 has been observed on murine and human macrophages following *in vitro* stimulation with IFN-γ ± lipopolysaccharide (LPS), suggesting an association between CD38 and the pro-inflammatory M1 state (21, 22). However, *in vivo* expression of CD38 by tissue-resident macrophages remains to be demonstrated, and *in vitro* cultures may not represent *in vivo* conditions accurately. Considering the known correlation between TAMs and poor prognosis in HCC (6–9), this requires further investigation.

In the present study, we sought to ascertain the *in vivo* expression of CD38 on macrophages from tumor tissues obtained from patients with HCC. Using immunohistochemistry (IHC) and Multiplex immunofluorescence (mIF), we confirmed the co-expression of CD38 with the macrophage-specific marker CD68. Notably, through Kaplan-Meier survival analysis and multivariate Cox regression, we established that the presence of CD38^+^CD68^+^ macrophages predicted improved prognosis after surgery, while total CD68^+^ macrophage density was associated with poor prognosis. *In vitro* functional studies using THP-1 derived macrophages revealed CD38 expression to be associated with the M1 state, characterized by CD80 expression and secretion of TNFα and IL-6, all of which contribute to the anti-tumor immune response. Using the DEPArray™, an automated platform that is able to identify and recover single cells with high resolution and purity, we visualized the expression of CD38 on macrophage-like cells isolated from HCC tumors. Finally, using flow cytometry, the co-expression of CD38 with CD14^+^HLA-DR^+^ cells was further confirmed.

## Materials and Methods

### Patients and Tumor Samples

A total of 66 archival formalin-fixed, paraffin-embedded (FFPE) specimens from patients diagnosed with HCC between January 2001 and December 2011 at the Department of Anatomical Pathology, Division of Pathology, Singapore General Hospital, were analyzed. All samples were obtained prior to chemo- or radiotherapy. Clinicopathological parameters, including patient age, tumor size, histologic growth pattern, grade, subtype, lymphovascular invasion and axillary lymph node status, were reviewed (Supplementary Table 1). Tumors were staged and graded according to the AJCC staging system (23) and the Edmonson-Steiner grading system (24). Ishak fibrosis scoring (25, 26) was adopted to evaluate the fibrosis status of the non-neoplastic liver, as documented in the pathological diagnostic reports. The Centralized Institutional Review Board of SingHealth provided ethical approval for the use of patient materials in this study (CIRB ref: 2009/907/B).

### Tissue Microarray Construction

Tumor regions for tissue microarray (TMA) construction were selected based on pathological assessment, with >50% of the sample representing tumor area. For each sample, two or three representative tumor cores of 1 mm diameter were transferred from donor FFPE tissue blocks to recipient TMA blocks using a MTA-1 Manual Tissue Arrayer (Beecher Instruments, Inc., Sun Prairie, WI, USA).

### Multiplex Immunofluorescence Analysis of TMAs

Multiplex immunofluorescence (mIF) was performed using an Opal Multiplex fIHC kit (PerkinElmer, Inc., Waltham, MA, USA) as previously described by our group and other studies (20, 20, 27–36), on FFPE tissue sections. TMA sections of 4 μm thickness were incubated with primary antibodies against CD38 and CD68, followed by appropriate secondary antibodies (Supplementary Table 2). Following this, a fluorophore-conjugated tyramide signal amplification buffer (PerkinElmer, Inc.) was applied, and DAPI was used as a nuclear counterstain. Images were acquired using a Vectra 3 pathology imaging system microscope (PerkinElmer, Inc.) and analyzed using inForm software (version 2.4.1; PerkinElmer, Inc.) (28, 37, 38). Maximally selected rank statistics (39) were applied using the maxstat R package to find optimal cut-off points for variables with good survival outcome prediction (Cutoff for CD38^+^ macrophages = 56.9%, whereas cutoff for CD68 immune infiltrates = 19.2%).

### *In vitro* THP-1 Functional Study

THP-1 cells were cultured and polarized based on a previously published protocol (21). In brief, cells were cultured in complete RPMI 1640 (Gibco; Thermo Fisher Scientific, Inc.) supplemented with 10% fetal bovine serum (FBS) (Gibco), 1% penicillin/streptomycin (Gibco), and 50 μM 2-mercaptoethanol (Gibco). Cells were then plated in 24-well plates at a density of 5 × 10^5^ cells/ml, differentiated with 50 ng phorbol myristate acetate (Sigma-Aldrich) for 24 h, washed, and cultured in fresh medium for 48 h. Macrophages were polarized to the M1 state with 20 ng/ml IFN-γ (R&D) and 100 ng/ml LPS (Sigma-Aldrich), or M2 state with 20 ng/ml IL-4 (R&D). After 24 h, cells were harvested for analysis by flow cytometry. Culture supernatant was collected for analysis of cytokines by ELISA.

### ELISA

ELISA was performed to detect IL-6, TNFα, IL-10, EBI3 (IL-35 subunit), and IL-12(p70). IL-6, TNFα and IL-10 kits were purchased from eBioscience (San Diego, CA, USA); EBI3 kit was purchased from R&D Systems, Inc. (Minneapolis, MN, USA); IL-12(p70) kit was purchased from RayBiotech (Norcross, GA, USA). Wells of microtiter plates were coated (18 h, room temperature) with respective capture antibody in 100 μl of coating buffer and were then blocked with 1% BSA in PBS (reagent diluent) for 1 h at room temperature. Samples or standards (100 μl) were loaded in duplicates and incubated for 2 h at room temperature, followed by the addition of 100 μl detection antibody for additional 2 h at room temperature. HRP-conjugated with Strepavidin (1:20,000) in reagent diluent was added (20 min, room temperature) and the reaction was visualized by the addition of 50 μl chromogenic substrate (TMB) for 10 min. The reaction was stopped with 100 μl H_2_SO_4_ and absorbance at 450 nm was measured using ELISA microplate reader (Perkin Elmer's Enspire 2300). Plates were washed three times with washing buffer (PBS, pH 7.4, containing 0.05% (v/v) Tween 20) after each step. As a reference for quantification, individual standard curves were established by serial dilution of respective recombinant cytokines.

### Tissue Dissociation and Isolation of Leukocytes

Liver tissue was cut into fine pieces and digested with 0.5 mg/ml Collagenase IV (Gibco; Thermo Fisher Scientific, Inc., Waltham, MA, USA) and 0.05 mg/ml DNAse I (Sigma-Aldrich; Merck KGaA, Darmstadt, Germany) in complete RPMI (Gibco; Thermo Fisher Scientific, Inc.) for 30 min at 37°C. Digested tissue was filtered using a 70 μm cell strainer. Cells were pelleted and treated with red blood cell lysis buffer (G-Biosciences, St Louis, MO, USA) for 5 min at room temperature. Cell debris was removed using Debris Removal Solution (Miltenyi Biotec, Ltd., Bergisch Gladbach, Germany) and tissue-infiltrating leukocytes were subsequently isolated using CD45 (TIL) MicroBeads, Human (Miltenyi Biotec, Ltd.), according to the manufacturer's protocol.

### Flow Cytometry

Cells were incubated with Fixable Viability Dye eFluor™ 455UV (eBioscience; Thermo Fisher Scientific, Inc.) for 30 min at 4°C for live/dead discrimination. Fc receptors were blocked using Human TruStain FcX™ (BioLegend; San Diego, CA, USA) for 5 min at room temperature. Cell surfaces were labeled with antibodies against markers of interest (refer to Supplementary Table 3 for antibody panel) for 30 min at 4°C. Matched isotype controls were included for antibodies against CD38. Single color compensation controls were prepared using Ultracomp eBeads (eBioscience; Thermo Fisher Scientific, Inc.). Samples were read in an LSRFortessa™ flow cytometer (BD Biosciences, Franklin Lakes, NJ, USA). Data analysis was performed using FlowJo^®^ V10 software (FlowJo LLC, Ashland, OR, USA).

### DEPArray™

Dissociated leukocytes were Fc-blocked using Human TruStain FcX™ (BioLegend; San Diego, CA, USA) and their surfaces labeled with antibodies against CD45, CD14, and CD38 (refer to Supplementary Table 3 for antibody panel). Cells were fixed with PFA and washed with SB115 buffer (Menarini Silicon Biosystems, Castel Maggiore, Italy). Approximately 10,000 cells were loaded into the DEPArray™ cartridge (Menarini Silicon Biosystems) for imaging, and were visually inspected to confirm the expression of markers of interest.

### Validation, follow-Up, and Statistical Analysis

Follow-up data were obtained from medical records. Disease-free survival (DFS) and overall survival (OS) were defined as the time from diagnosis to recurrence or death/date of last follow-up, respectively. Statistical analysis was performed using RStudio 1.1.456 running R 3.5.0 (40, 41) (R-core Team, R Foundation for Statistical Computing, Vienna, Austria) and SPSS 23.0 for Windows (IBM Corp., Armonk, NY, USA). Multivariate Cox regression was performed to evaluate the effect of selected variables on survival after adjusting for clinicopathological parameters, including for tumor size and stage. P < 0.05 indicates a statistically significance difference.

## Results

### Expression of CD38 by Tumor-Associated Macrophages (TAMs) in HCC Is Visualized by Immunohistochemistry (IHC)

**Figure 1:**
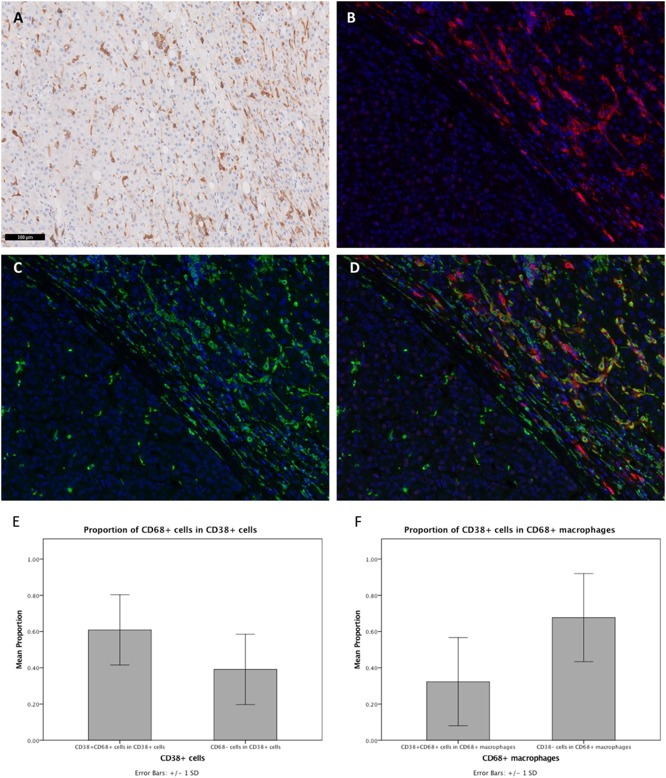
Expression of CD38 by TAMs from HCC patients was visualized by IHC. **(A)** Conventional IHC staining of a representative FFPE tumor section revealing the presence of macrophage-like cells (brown) in the liver sinusoids. **(B,C)** Immunofluorescent staining for CD38 (red) and macrophage marker CD68 (green), respectively, on tumor section. **(D)** Merged image showing co-localization of CD38 (red) and CD68 (green) in the tumor microenvironment. Images are presented at 40× magnification. **(E)** Proportions of CD38^+^CD68^+^ and CD38^+^CD68^−^ leukocytes. **(F)** Proportions of CD38^+^CD68^+^ and CD38^−^CD68^+^ macrophages. Data is presented as the mean ± standard deviation.

IHC staining of FFPE tumor sections revealed the expression of CD38 by cells residing in the liver sinusoids. As shown in Figure 1A, cells that were stained had an irregular shape with many cytoplasmic extensions. We therefore hypothesized that these CD38-expressing cells may be macrophages, based on both their morphology and anatomical location (42). Moreover, previous studies have demonstrated that CD38 is expressed on macrophages in animal models (13, 22, 43, 44) and that CD38 expression on macrophages is correlated with inflammation in humans (21). Thus, we performed Opal-Vectra multiplex IHC on the specimens, using protocols previously optimized and reported by our group and other laboratories (20, 27, 30–35), to identify the co-expression of CD68 and CD38 in liver immune cells. CD38 and CD68 were frequently co-expressed by the same cells (Figures 1B–D), with 62.9 ± 19.2% of CD38^+^ cells bearing the macrophage marker CD68 and 32.3 ± 24.1% of CD68^+^ cells being CD38^+^ (Figures 1E,F). This suggested that a large proportion of CD38^+^ cells were macrophages.

### High CD38^+^ Macrophage Density Is Associated With Improved Survival in HCC

**Figure 2:**
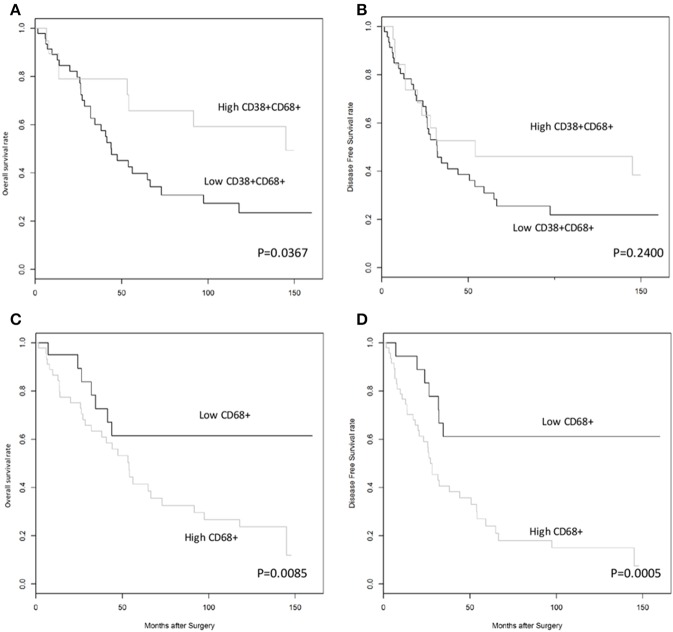
High density of CD38^+^, but not total, CD68^+^ TAMs was associated with improved overall survival rates in patients with HCC. **(A,B)** Kaplan-Meier curves showing overall and disease free survival rates, respectively, for patients grouped according to high or low density of CD38^+^CD68^+^ macrophages in the tumor microenvironment. **(C,D)** Kaplan-Meier curves showing overall and disease free survival rates, respectively, for patients grouped according to high or low density of CD68^+^ total macrophages in the tumor microenvironment. Cut-offs for high/low density of CD38^+^CD68^+^ macrophages (0.5690) and CD68^+^ total macrophages (0.1918) were based on optimal values calculated using statistical software.

Next, we investigated whether CD38^+^ macrophage density in tumors affected survival outcome in patients with HCC. Patients with high density of CD38^+^CD68^+^ macrophages experienced significantly improved overall survival (OS) compared with those with low density of CD38^+^CD68^+^ macrophages (*P* = 0.0367; Figure 2A); in contrast, disease free survival (DFS) did not differ significantly between groups (*P* = 0.2400; Figure 2B). Multivariate analysis further supported the association between a high density of intratumoral CD38^+^ macrophages and significantly improved OS (HR = 0.41; 95% CI 0.18–0.93; *P* = 0.0322; Table 1).

**Table 1:** Multivariate analysis of the effect of intratumoral macrophage density on OS and DFS, adjusted for tumor size, grade, age, and lymph node status.

**Biomarkers**	**HR**	**95% CI**	***P*-value**
**OS**			
**Proportion of intratumoral CD38^**+**^ macrophages in HCC**	0.41	0.18–0.93	
High vs. low			0.0322^*^
**CD68^**+**^ immune infiltrates**	4.11	1.76–9.59	
High vs. low			0.0011^*^
**DFS**			
**Proportion of intratumoral CD38^**+**^ macrophages in HCC**	0.62	0.31–1.24	
			0.1769
High vs. low			
**CD68 immune infiltrates**	3.12	1.34–7.27	
High vs. low			0.0085^*^

* *Bold values indicate statistically significant*.

The effect of total CD68^+^ macrophages on prognosis was also investigated. Notably, high CD68^+^ macrophage density was associated with reduced OS (HR = 3.12; 95% CI 1.34–7.27; *P* = 0.0085) and DFS (HR = 4.44; 95% CI 1.92–10.26; *P* = 0.0005) in HCC patients (Figures 2C,D, respectively). Multivariate analysis also revealed that CD68^+^ macrophage density was an independent prognostic marker for reduced OS (HR = 3.01; 95% CI 1.27–7.16; *P* = 0.0124) and DFS (HR = 4.11; 95% CI 1.76–9.59; *P* = 0.0011) after adjusting for clinicopathological parameters (Table 1).

### CD38 Up-regulation Was Associated With M1 Macrophages, Characterized by Expression of Co-stimulatory CD80 and a Pro-inflammatory Cytokine Profile

**Figure 3:**
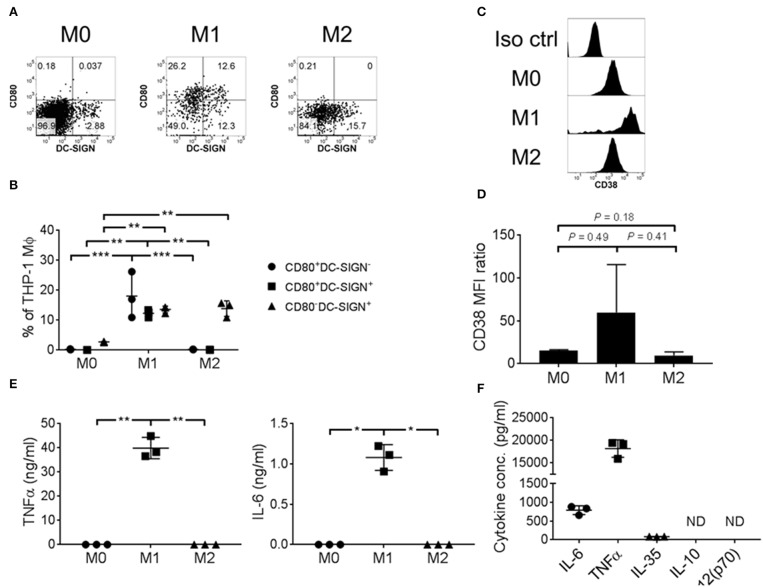
THP-1 derived M1 macrophages expressed CD38 and secreted pro-inflammatory cytokines. THP-1 cells were polarized into M0/M1/M2 macrophages with differential surface expression of **(A,B)** CD80 and/or DC-SIGN; **(C,D)** CD38. Representative flow cytometry data are shown. MFI: geometric mean fluorescent intensity. **(E)** IL-6 and TNFα cytokine secretion, as determined by ELISA using culture supernatant. The data represents the means ± s.d. **(F)** Cytokine secretion profile of M1 macrophages, as determined by ELISA using culture supernatant. ND: not detected. ^*^P ≤ 0.05; ^**^P ≤ 0.01; ^***^P ≤ 0.001.

To ascertain the function of CD38^+^ macrophages, we took advantage of the THP-1 cell line to investigate surface expression and cytokine secretion of CD38^+^ macrophages under *in vitro* culture conditions. THP-1 cells were polarized into M1/M2 macrophages that showed differential expression of CD80 and DC-SIGN (Figures 3A,B), consistent with the results of a previous study (45). Notably, robust expression of CD80 was observed on M1 macrophages but not M0 or M2. Additionally, we found up-regulation of CD38 on M1 macrophages (Figures 3C,D), concomitant with pro-inflammatory IL-6 and TNFα secretion (Figure 3E). By contrast, M0 or M2 macrophages did not secrete IL-6 or TNF-α (Figure 3E). As compared to IL-6 (786.7 ± 117.1 pg/ml) and TNFα (18.2 ± 1.9 ng/ml), the level of IL-35 secretion was minimal (82.0 ± 4.2 pg/ml) and there was no detectable IL-10 and IL-12(p70) secretion from M1 macrophages (Figure 3F). Altogether, our results suggest that CD38^+^ macrophages are associated with an M1 phenotype that is characterized by higher CD80 expression and a pro-inflammatory cytokine profile.

### Confirmation of CD38 Expression on Macrophages by DEPArray™ and Flow Cytometry

**Figure 4:**
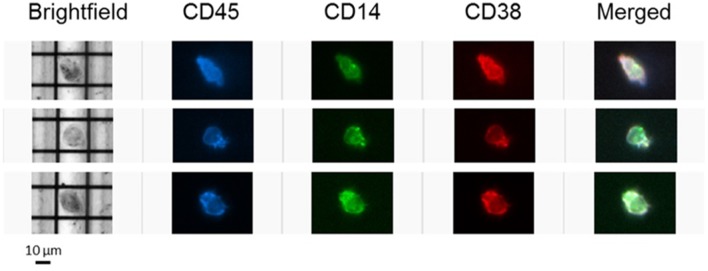
CD38-expressing tumor-infiltrating leukocytes were visualized using the DEPArray™ platform. Three single cells resembling macrophages are presented. Cells are large (>10 μm), irregularly shaped and express myeloid marker CD14.

**Figure 5:**
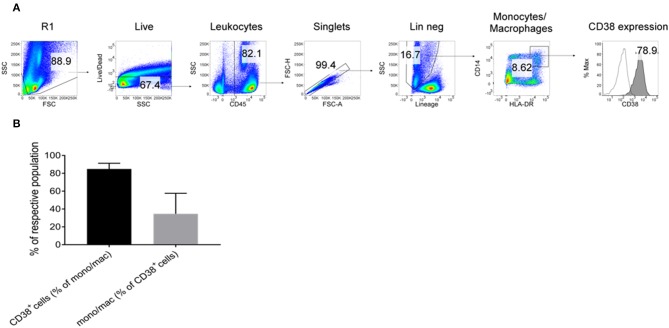
Expression of CD38^+^ by tumor-infiltrating monocytes/macrophages further confirmed by flow cytometry. **(A)** Tumor-infiltrating leukocytes from four HCC patients were analyzed. Lineage^+^ cells (CD3/CD7/CD19/CD20/CD56) were excluded in a dump channel. Monocytes/macrophages were identified by co-expression of CD14 and HLA-DR. Expression of CD38 (filled histogram) was determined with respect to the isotype control antibody (clear histogram). **(B)** % of monocytes/macrophages that expressed CD38 (black column) and % of CD38^+^ leukocytes that were monocytes/macrophages (gray column). The data represents the means ± s.d.

To further confirm the expression of CD38 on TAMs from patients with HCC, tumor-infiltrating leukocytes (TILs) were examined using the DEPArray™, which is an automated platform capable of visualizing and isolating single cells with high resolution and purity. Images captured by the DEPArray™ revealed the presence of large (>10 μm), irregularly shaped CD45^+^CD14^+^ cells that resembled macrophages (Figure 4). CD38 expression was clearly detected on the surfaces of these cells. Next, flow cytometry was performed to analyse TILs in greater detail. Monocytes/macrophages were identified in tumor tissues from four HCC patients using the gating strategy shown in Figure 5A. The vast majority of CD14^+^HLA-DR^+^ cells expressed CD38 (84.9 ± 7.4%; Figure 5B), indicating enrichment of CD38^+^ monocytes/macrophages in the tumors of these four patients. In terms of total CD38-expressing leuckocytes, monocytes/macrophages constituted only 34.6 ± 26.6% (Figure 5B), indicating the presence of other CD38-expressing immune lineages.

## Discussion

Through multiplex IF and conventional IHC, our group was able to identify and enumerate CD68^+^ TAMs present in the tumor samples from HCC patients. We established that CD68^+^ TAM density was correlated with poor prognosis in HCC, in line with the finding of a previous report (6). The tumor microenvironment is known to be dominated by cytokines and growth factors that establish a Th2-type, anti-inflammatory immune environment that favors the survival of tumor cells. Under such environmental influences, TAMs are driven toward a M2 phenotype. This transition is accompanied by increased pro-tumoral activities, including the suppression of the adaptive immune response and the promotion of cancer proliferation, angiogenesis and extracellular matrix remodeling (46).

To the best of our knowledge, the present study represents the first instance that CD38 expression has been demonstrated on human macrophages in an *in vivo* setting. Previously, CD38 expression was reported on macrophages isolated from mice (13, 47), on a murine macrophage cell line (48) and on human macrophages under *ex vivo* conditions (21). We initially observed the co-localization of CD38 and CD68 signals in the sinusoids of liver sections, and subsequently from freshly isolated TILs. CD38 was visually confirmed on macrophage-like CD14^+^ cells using DEPArray™, and also on CD14^+^HLA-DR^+^ monocytes/macrophages using flow cytometry. Notably, statistical analysis of the IHC data revealed that CD38^+^CD68^+^ macrophage density was positively associated with improved prognosis, in direct contrast to total CD68^+^ macrophage density. This suggested the existence of functionally-distinct macrophage subsets, with CD38 marking a subpopulation of pro-inflammatory macrophages (21) that may play a role in tumor suppression. Supporting this hypothesis was data from our *in vitro* functional study of THP-1 cells polarized to M1 or M2 macrophages. Specifically, M1 macrophages were distinguished by higher expression of CD80—a costimulatory molecule that signals through CD28 to amplify T cell activation and contributes to anti-tumor immunity (49, 50). We also found that CD38 was up-regulated on M1 macrophages, concomitant with a pro-inflammatory cytokine profile characterized by robust IL-6 and TNFα secretion, with minimal IL-35 secretion and no secretion of IL-10 and IL-12 cytokines. By contrast, M0 or M2 macrophages did not produce detectable levels of all cytokines examined. A previous study reported that deletion of IL6 or TNFα in a mouse model of HCC accelerated tumor development (51). Altogether, our results suggest that CD38^+^ macrophages are associated with an M1 phenotype that is characterized by higher CD80 expression and a pro-inflammatory cytokine profile, all of which contribute to the anti-tumor immune response. This effect may account for the improved prognosis of HCC patients with higher proportion of tumor-infiltrating CD38^+^ macrophages. To further appreciate the complexities of TAMs in liver cancer, a future single cell study could be conducted. In view of this, the DEPArray™ platform may be useful for isolating single cells with high purity, as required for sensitive downstream genomic and expression analyses (52–54).

It is important to note that CD38 is involved in the production of adenosine from NAD^+^. Adenosine is known to be an inhibitor of effector T cells and is produced by various regulatory cells expressing CD38, including myeloid-derived suppressor cells, mesenchymal stem cells and NK cells (55). In view of this, the expression of CD38 on tumor-infiltrating macrophages in the present study raises the possibility of participation in this mode of immunosuppression. This suggests the presence of a complex interplay between the inflammatory response and immune suppression via adenosine production.

When flow cytometry was used to investigate TILs from four patients with HCC, high levels of CD38 expression were found among the monocytes/macrophages. However, these monocytes/macrophages represented only 34.6% of total CD38^+^ leukocytes, which indicated the presence of other CD38^+^ immune lineages. In our previous study concerning TILs in HCC, we reported expression of CD38 on lymphocytes, NK cells, NKT cells and monocytes (20). Such broad expression was in line with the reported ubiquity of CD38 across the immune system (10).

Of interest to us in future studies is CD157, a paralogue of CD38 derived from gene duplication. CD157 also possesses dual receptorial and NADase functions and is widespread across lymphoid tissues, including macrophages (56). Given the high level of sequence similarity between CD38 and CD157, it is important to establish whether CD157^+^ macrophages also predict improved prognosis in patients with HCC.

In conclusion, this study confirmed the expression of CD38 by human macrophages *in vivo*, and established that CD38^+^ macrophages are correlated with improved prognosis after surgery. The function of CD38 in myeloid cells warrants further study, and this marker may be utilized in routine diagnostic work in the era of cancer immunotherapy.

## Ethics Statement

The Centralized Institutional Review Board of SingHealth provided ethical approval for the use of patient materials in this study (CIRB ref: 2009/907/B).

## Author Contributions

JY, TSL and TKL conceived and directed the study. JY and TSL supervised the research. JHL, HN, and CL collated and interpreted the data and performed biostatistical analysis. XS and JL constructed TMAs and performed IHC. HL performed additional biostatistical analysis. HN, XS, and J-KK performed immunohistochemical scoring. FM, KS, CO, TL, WL, and VC contributed to the scientific content of the study. SC and HT provided scientific inputs from Oncology perspectives. SL and CC provided scientific inputs from Surgery perspectives. HN, JHL, and JY drafted the manuscript with the assistance and final approval of all authors.

### Conflict of Interest Statement

JHL and TSL are employees of A. Menarini Biomarkers Singapore Pte Ltd. FM received research support from Janssen Pharmaceuticals, Celgene, Tusk Therapeutics and Centrose, and served on advisory boards for Centrose, Tusk Therapeutics, Jenssen, Takeda and Sanofi. The remaining authors declare that the research was conducted in the absence of any commercial or financial relationships that could be construed as a potential conflict of interest.
