# Supplementary material for: Expression of CD38 on Macrophages Predicts Improved Prognosis in Hepatocellular Carcinoma
Source: Front Immunol. 2019 Sep 4;10:2093. doi: 10.3389/fimmu.2019.02093 (PMC6738266; doi:10.3389/fimmu.2019.02093)
Supplement: Supplementary file 1 [file Table_1.DOCX]

Supplementary Material

# Supplementary Tables

**Supplementary Table 1.** Clinicopathological characteristics of patients analysed for mIF fluorescence

| Clinicopathological characteristics | Frequency (proportion) |
| --- | --- |
| Age (years) | |
| ≤55 | 17 (25.8%) |
| >55 | 49 (74.2%) |
| Ethnicity | |
| Chinese | 55 (83.3%) |
| Indian | 1 (1.5%) |
| Malay | 2 (3.0%) |
| Others | 8 (12.1%) |
| Size (mm) | |
| ≤20 | 64 (97.0%) |
| >20 | 2 (3.0%) |
| Tumor histological grade* | |
| 1 | 12 (18.5%) |
| 2 | 26 (40.0%) |
| 3 | 27 (41.5%) |
| Pathological stage ^ | |
| I | 42 (63.6%) |
| II | 13 (19.7%) |
| III/IV | 11 (14.7%) |

* Graded according to the 4-scale Edmondson and Steiner grading system (24).
^ Staged according to the AJCC staging system (23).

**Supplementary Table 2.** List of antibodies used for multiplex immunofluorescence.

| **Antibody** | **Clone** | **Dilution** | **Source** | **Labeling pattern** |
| --- | --- | --- | --- | --- |
| DAPI | N/A | 1 drop : 500µl | Perkin Elmer (FP1490) | Cell nucleus |
| CD38 | SPC32 | 1:50 | Novo Castra (NCL-CD38-290) | Immune cells, Cell membrane |
| CD68 | PGM1 | 1:100 | Dako (M0876) | Immune cells, cytoplasm |

**Supplementary Table 3.** List of antibodies used for flow cytometry and DEPArray^TM^.

| **Antibodies for flow cytometry** | | | | | | | |
| --- | --- | --- | --- | --- | --- | --- | --- |
| # | **Antigen** | **Fluorophore** | **Clone** | **Manufacturer** | **Catalogue number** | **Working dilution** | **Remarks** |
| 1 | CD45 | APC | HI30 | BioLegend | 304012 | 1:20 |  |
| 2 | CD3 | FITC | UCHT1 | BioLegend | 300406 | 1:40 | Lineage dump |
|  | CD7 | FITC | 4H9 | eBioscience | 11-0078-42 | 1:40 |  |
|  | CD19 | FITC | HIB19 | BioLegend | 302206 | 1:40 |  |
|  | CD20 | FITC | 2H7 | BioLegend | 302034 | 1:40 |  |
|  | CD56 | FITC | MEM-188 | BioLegend | 304604 | 1:40 |  |
| 3 | CD14 | ECD | RM052 | Beckman Coulter | IM2707U | 1:40 |  |
| 4 | HLA-DR | BV785 | L243 | BioLegend | 307642 | 1:20 |  |
| 5 | CD38 | PE | HIT2 | BioLegend | 303506 | 1:20 |  |
| 6 | CD80 | BV650 | 2D10 | BioLegend | 305227 | 1:20 |  |
| 7 | DC-SIGN | APC/Fire 750 | 9E9A8 | BioLegend | 330115 | 1:20 |  |
| **Antibodies for DEPArray^TM^** | | | | | | | |
| # | **Antigen** | **Fluorophore** | **Clone** | **Manufacturer** | **Catalogue number** | **Working dilution** | **Remarks** |
| 1 | CD45 | APC | HI30 | BioLegend | 304012 | 1:20 |  |
| 2 | CD14 | AF488 | 63D3 | BioLegend | 367129 | 1:20 |  |
| 3 | CD38 | PE | HIT2 | BioLegend | 303506 | 1:20 |  |

APC, allophycocyanin; FITC, fluorescein isothiocyanate; ECD, phycoerythrin-Texas red; PE, phycoerythrin.
